# No Evidence That Hormonal Contraceptives Affect Chemosensory Perception

**DOI:** 10.1177/2041669520983339

**Published:** 2021-02-01

**Authors:** Martin Schaefer, Behzad Iravani, Artin Arshamian, Johan N. Lundström

**Affiliations:** Department of Clinical Neuroscience, 27106 Karolinska Institutet, Stockholm, Sweden; Department of Clinical Neuroscience, 27106 Karolinska Institutet, Stockholm, Sweden; Gösta Ekman Laboratory, Department of Psychology, Stockholm University, Stockholm, Sweden; Department of Clinical Neuroscience, 27106 Karolinska Institutet, Stockholm, Sweden; Department of Psychology, University of Pennsylvania, Philadelphia, United States; Stockholm University Brain Imaging Centre, Stockholm University, Stockholm, Sweden; Monell Chemical Senses Center, Philadelphia, Pennsylvania, United States

**Keywords:** oral contraceptives, chemosensory perception, Bayesian, olfaction, trigeminal, taste, the pill

## Abstract

The use of oral contraceptives (OC) in the form of a hormonal pill has been widespread for decades. Despite its popularity and long-time use, there is still much ambiguity and anecdotal reports about a range of potential side effects. Here, we addressed the potential effect of OC use on chemosensory perception. Previous research has almost exclusively focused on olfaction, but we expanded this to the trigeminal system and the sense of taste. We used Bayesian statistics to compare the olfactory, trigeminal, and taste detection abilities between a group of 34 normal cycling women and a group of 26 women using OC. Our results indicated that odor, trigeminal, and taste thresholds were not affected by the use of OC. Moreover, neither odor perception, nor taste perception was affected; all with Bayes factors consistently favoring the null hypothesis. The only exception to these results was odor identification where Bayes factors indicated inconclusive evidence. We conclude that effects of OC use on chemosensory perception are unlikely, and if present, likely are of no to little behavioral relevance.

The hormonal contraceptive pill is the most commonly used form of oral contraceptives OC; (Cooper & Adigun, 2018; United Nations, Department of Economic and Social Affairs, 2018). 

However, OC use is associated with a wide variety of unwanted side effects, such as nausea, menstrual bleeding irregularities, mood changes, reduced sexual interest, among others; all of which can lead to discontinuation (Khan, 2003; Moreau et al., 2007; Rosenberg & Waugh, 1998; Sanders et al., 2001). While some of the side effects are confirmed, some might be propagated by hearsay and the power of suggestion (Grimes & Schulz, 2011), and others might originate from biased results, common among research disciplines (Ioannidis, 2005). Specifically, the well-known publication bias leading to an inflation of false positive findings (Ferguson & Heene, 2012) may have skewed the results so that potential side effects of OC use are exaggerated. Given OC effectiveness as a family planning tool and that discontinuation is associated with an increase in unwanted pregnancies (Rosenberg et al., 1995), this would be unfortunate. Thus, it is important that discontinuation of OC is based on well-grounded facts.

One potential side effect of OC mentioned in the literature is a changed olfactory perception. For example, higher olfactory performance (assessed by “TDI score,” which characterizes the individual olfactory performance as the sum of odor threshold, discrimination, and identification ability; Hummel et al., 1997; Kobal et al., 1996) was negatively related to the dosage of ethinyl estradiol in the pill (Kollndorfer et al., 2016). In contrast, olfactory performance has also been positively related to the duration of OC intake (Derntl et al., 2013). On the other hand, OC has been linked to lower olfactory sensitivity for musk-like odors (as indicated by higher detection threshold levels; Caruso et al., 2001; Renfro & Hoffmann, 2013), as well as lower sensitivity for social odors, but simultaneously higher sensitivity for environmental odors (Lundström et al., 2006). Also, OC seems to change partner body odor preference in women, shifting it from preferring the odor of men with a different major histocompatibility complex than oneself to men with a more similar major histocompatibility complex (Allen et al., 2019; Roberts et al., 2008; Wedekind et al., 1995). Similarly, withdrawal of OC has been linked to reduced discrimination for body odors, whereas no effects were found for ordinary odorants (Endevelt–Shapira et al., 2020). Because it has been demonstrated that olfactory perception fluctuates over the course of the menstrual cycle, and OC works by introducing exogenous hormones to interfere with this tightly orchestrated hormonal sequence, there is a well-grounded concern, as well as a potential mechanism, for OC to interfere with olfactory performance (Grillo et al., 2001; Lundström et al., 2006; Martinec et al., 2014). However, as demonstrated earlier, studies examining the effect of the hormonal contraceptive pill on olfactory perception have yielded mixed and sometimes contradictory results.

There are a number of potential reasons for these contradicting findings regarding OC use and olfactory perception. For example, the odorants tested across these studies varies widely. Because olfactory perception is affected by the trigeminal system (Frasnelli et al., 2007), variation across studies might arise from differences in odor trigeminality. Unfortunately, it is unknown if OC impact the trigeminal function. Whether taste is affected by OC is also mostly unknown, with one study claiming to have found a preference for sucrose among users of OC with a low progestin content, and no recent studies (Dippel & Elias, 1980). A potential indication that the trigeminal sense and taste might be affected by OC use comes from studies with pregnant women, which are also in a state of altered hormonal homeostasis, and where small differences have sometimes been found compared with nonpregnant women (Bhatia & Purl, 1991; Duffy et al., 1998; Nordin et al., 2004, 2005; Olofsson et al., 2005). Previous reviews that have tried to disentangle the large amount of published reports and anecdotal evidence about different hormonal stages (sex differences, hormonal contraception, and pregnancy) and olfactory perception found that support for simple associations between hormone levels and olfactory function is generally lacking (Cameron, 2014; Doty & Cameron, 2009). The higher odds of statistically significant results to be published further increases the difficulty to provide clarity on whether OC use affects chemosensory perception (Dwan et al., 2008).

Here, we set out to asses OC effects on chemosensory functions more thoroughly by targeting not only odor perception but trigeminal and taste perception as well.We compared odor discrimination, odor identification, and detection threshold levels for odor, trigeminal, and taste stimuli, as well as taste perception between normal cycling (NC) women and OC users. We analyzed these tests using a Bayesian approach to enable the assessment of the null hypothesis.

## Methods

### Participants

Sixty women in the age range of 18–35 years (mean age 25, standard deviation [*SD*] ± 4.0) participated in the study and provided written, informed consent. All aspects of the study were approved by the University of Pennsylvania’s Institutional Review Board. Parts of this data set have previously been used for other unrelated questions (Lundström et al., 2012).

All participants were in good general health, were not currently taking any prescription medication—with the exception of hormonal contraceptives (see later), and did not knowingly suffer from any endocrine, neurological, or autoimmune diseases. Additional exclusion criteria consisted of being an active smoker or having suffered a head trauma with loss of consciousness. Participants did not wear any perfume or scented products on the day of testing, had not smoked, nor did they chew gum, eat, or drink anything but water 1 hour prior to testing.

Twenty-six of the participants were using monophasic or biphasic OC. All the women not using OC (freely circulating, *n* = 34) had a naturally regulated menstrual cycle of normal range (range: 26–33 days). To control for potential menstrual cycle effects, 14 of the freely circulating women were tested in the follicular phase of their menstrual cycle (Day 7–15; mean 9.7, *SD* ± 1.1), and 14 were tested in their luteal phase (Day 16–26; mean 20.8, *SD* ± 2.8), as defined by postmenses onset based on self-report (Lundström et al., 2006). Six freely circulating women and three woman taking OC were tested during their menses phase (Day 1–6; mean 3.7, *SD* ± 1.6).

### Chemosensory Tasks

#### Odor Discrimination Task

Odor discrimination ability was assessed with the Sniffin’ Stick odor discrimination test (Hummel et al., 1997). In this test, subjects are presented with 16 triplets of felt-tipped odorized pens. Every time the subject is presented with three odorized pens in a row of which two contain the same odor and one is different. The subject then chooses which of the three pens contains the different odor, allowing for a maximum score of 16 and with a chance level score of 5.

#### Odor Identification Task

Odor identification ability was tested with the Monell Extended Sniffin’ Sticks Identification Test (MONEX-40; Freiherr et al., 2012; Hummel et al., 1997). This test consists of presenting subjects with 40 felt-tipped odorized pens and an accompanying card with the name of four objects on it. The participant then chooses which of the four named objects corresponds to the odor just presented, allowing for a maximal score of 40 and a chance level score of 10.

#### Detection Threshold Tasks for Odor, Trigeminal, and Taste Stimuli

The odor, trigeminal, and taste detection threshold tasks consisted of presenting subjects with a range of stimuli of increasing intensity (concentration) and blank stimulus/stimuli. The task was to identify the stimulus containing the odorant/tastant/trigeminal substance. The detection thresholds were assessed using a three-alternative, forced-choice, ascending staircase paradigm (Wetherill & Levitt, 1965). The staircase was reversed when subjects correctly identified the odor, trigeminal, or taste stimuli in two successive trials, with a subsequent reversal of the staircase upon failure to correctly identify the odor. Depending on the task, five or seven reversals were collected this way (outlined later), and the mean of the last four reversals served as the threshold estimate. The duration of the interstimulus intervals was 20 seconds.

##### Odors

Olfactory detection thresholds were measured for n-butanol (CAS 71-36-3; unless noted otherwise, all chemicals used in the study were obtained from Sigma-Aldrich) and peanut oil (TAK-053887; Takasago Corporation). We chose these two odors to control for differences in chemical composition (monomolecular, complex mixture, respectively) and ecological relevance (“chemical odor”, food-associated odor, respectively).Sixteen different concentrations of n-butanol diluted in odorless 1,2 propanediol (CAS 57-55-6) were prepared ranging from 4% volume/volume (v/v) to 1.2 × 10^−4^% v/v in twofold dilution steps. The peanut oil was diluted in odorless silica-filtered, light mineral oil (CAS 8042-47-5) to 16 different concentrations ranging from 17.5% v/v to 2.6 × 10^−3^% v/v in 1.8-fold dilution steps. A pilot study (*n* = 20) showed that these concentration ranges capture the threshold of 19 out of 20 normosmic subjects. Participants were blindfolded and picked the odor-containing bottle out of three bottles that were presented to them in a randomized order, in each dilution step. One of the bottles contained the diluted odorant, and the two others contained the solvent. Each subject started with the weakest odor concentration and worked their way up the concentration ladder until they successfully identified the stimuli in two successive trials, upon which the staircase was reversed and the odorant concentrations were lowered again. Seven staircase reversals were collected, and the maximum possible score was 16.

##### Trigeminal Stimuli

Because humans can only lateralize chemical vapor when concentrations reach high enough levels to elicit a trigeminal sensation, and not intranasal presented odorants alone, a nasal lateralization task was used to assess trigeminal sensitivity (Kobal et al., 1989; Wysocki et al., 1997; but see Porter et al., 2005). Participants were presented with clean air into one nostril and odorized air into the other nostril simultaneously, after which they indicated which nostril received odorized air (spatial, two-alternative, forced-choice). Sixteen concentrations of l-menthol (menthol) crystals (CAS 2216-51-5) in 1,2 propanediol were created, ranging from 50% v/v to 1.71 × 10^−1^% v/v in 1.5-fold dilution steps, and filled into Teflon nosepiece covered bottles (for detailed description, see Wysocki et al., 2003). Each subject started with the weakest l-menthol concentration and worked their way up the concentration ladder until they successfully identified the stimuli in two successive trials, upon which the staircase was reversed and the l-menthol concentrations were lowered again. Seven staircase reversals were collected, and the maximum possible score was 16.

##### Taste Stimuli

Taste detection thresholds were measured for quinine monohydrochloride dehydrate (CAS 6119-47-7) and sucrose (CAS 57-50-1). These two compounds were chosen to cover a wide range of tastes (bitter, i.e., toxic signal, and sweet, i.e., nutrition signal, respectively). Both tastants were diluted in Millipore-filtered deionized water to 18 different concentrations in 1.25-fold dilution steps. The quinine concentrations ranged from 3.0 × 10^−5^ M to 6.8 × 10^−7^ M, and the sucrose concentrations ranged from 8.3 × 10^−2^ M to 1.9 × 10^−3^ M.

To exclude potential olfactory information from the decision-making process, participants wore noseclips during the taste detection task. Two cups were presented to the participants on each trial. One cup contained 10 ml of the tastant solution, whereas the other cup was filled with the clean diluent in equal amount. On each trial, the participant poured the entire content of a cup into their mouth, gently swirled it around for 10 seconds, and then spat it out and rinsed with deionized water before proceeding with the content of the second cup. Participants then selected which cup contained the tastant. Each subject started with the middle tastant concentration and worked their way up the concentration ladder until they successfully identified the stimuli in two successive trials, upon which the staircase was reversed and the tastant concentrations were lowered again. Five staircase reversals were collected, and the maximum possible score was 23.

#### Taste Perception Task

Participants further rated the tastants for perceived pleasantness, familiarity, intensity, and quality. Pleasantness and familiarity were rated on a visual analogue scale ranging from “extremely unfamiliar/unpleasant” to “extremely familiar/pleasant,” with “neutral” in the middle. Intensity and quality were rated on a labelled magnitude scale ranging from “no sensation” to “strongest imaginable,” with the steps “barely detectable,” “weak,” “moderate,” “strong,” and “very strong” in between. Different scales were used because the more cognitive perceptions of familiarity and pleasantness tend to scale linearly, whereas intensity and quality have a close link to physical stimuli and therefore scale logarithmically (Green et al., 1996).

The quinine was presented at a concentration of 3.0 × 10^−5^ M and the sucrose at a concentration of 0.047 M; both tastants were diluted in Millipore-filtered deionized water.

### Design

A between-subject design was employed, and testing order was pseudorandomized to limit carryover effects from odors to tastants, and vice versa. This design entailed that the test order was the same across participants, but the items within each test varied between participants in a predetermined manner. Demographic variables were collected in between threshold testing to allow recovery between threshold measurements and to limit adaptation and testing fatigue. Testing was performed in rooms specifically designed for chemosensory testing with high turnover of the room air, thus limiting the amount of residual odor, and dedicated taste spit sinks as well as deionized water taps were present. Total testing time, including frequent breaks, for each subject was approximately 3.5 hours.

### Statistics

The question of whether OC affects olfactory sensitivity has, to the best of our knowledge, previously only been investigated by using null hypothesis statistical testing. To enable model comparison between H0 versus H1, as well as enabling the quantification of evidence in favor of H0, we implemented Bayesian statistics using the JASP program to do the statistical analysis (JASP, 2018; Wagenmakers et al., 2018). Bayesian two-sample *t* test (Rouder et al., 2009) with a two-sided default Cauchy prior with location 0 and scale 0.707 was performed in JASP for the analysis of TDI, as well as trigeminal and taste thresholds. The results of the two odors and two tastants were combined into singular odor and taste outcomes by averaging them. Bayesian analysis of variance (ANOVA) with a default prior (*r* scale fixed effects = 0.5; *r* scale random effects = 1) default was performed to investigate the perception of tastants (Rouder et al., 2012). We used the default priors (as implemented in JASP) because they place mass in realistic ranges without being overcommitted to any one point (Rouder et al., 2009). This is important, as the default priors were chosen because of the ambiguous results of previous studies that have investigated the effect of OCs on olfactory sensitivity, with widely varying effect sizes (Caruso et al., 2001; Derntl et al., 2013; Kollndorfer et al., 2016; Landis et al., 2004), as well as the absence of studies on the effect of OC on taste and trigeminal sensitivity in humans. This type of priors has been shown to fit a large set of psychological data with moderate effect sizes (Rouder et al., 2009, 2012, 2016). Our interpretation of the Bayes factor (BF) follows standard recommendations (Jarosz & Wiley, 2014; Jeffreys, 1998). These state that a BF between 1 and 3 implies indecisive to anecdotal evidence, 3–10 substantial, and 10–30 strong evidence. For instance, BF_10_ = 4 indicates the data are four times more likely under H1 than under H0, whereas a BF_01_ = 4 would support H0 four times more than H1.

All figures were created in R with the ggplot2 package and were esthetically modified in Inkscape (Inkscape Project, 2018; R Core Team, 2018; Wickham, 2009).

## Results

**Figure 1.**
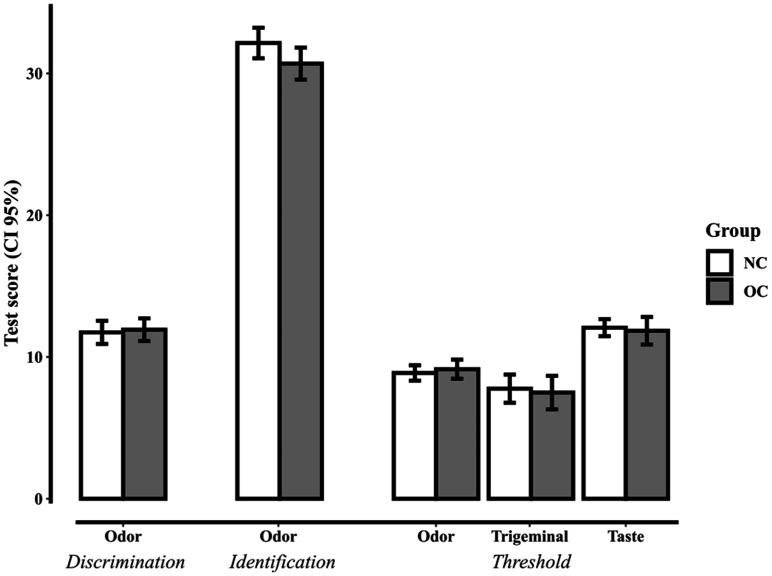
Mean scores of the different sensitivity tests for NC women and women using OC. Maximum possible scores were 16 for odor discrimination and odor and trigeminal threshold detection, 23 for taste threshold detection, and 40 for the odor identification task. Error bars display 95% CI. NC = normal cycling; OC = oral contraceptives; 95% CI = 95% confidence interval.

**Figure 2.**
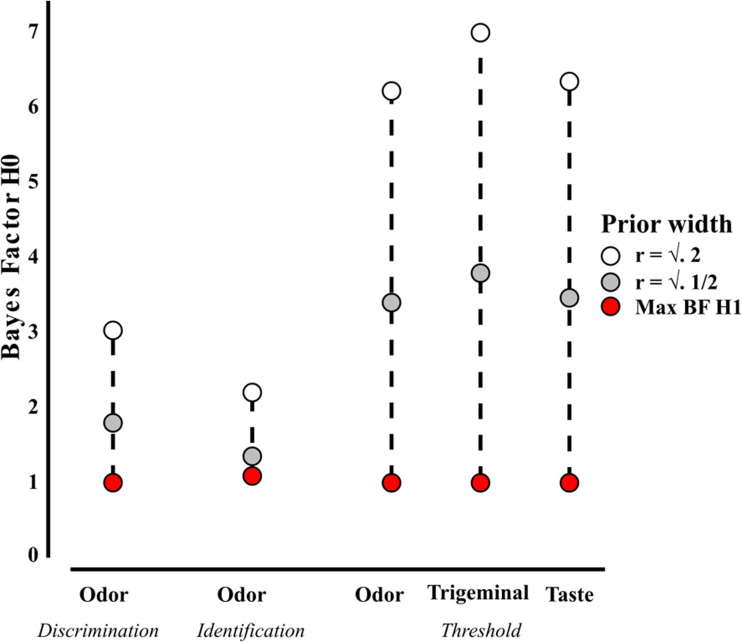
Summary of BF variation depending on prior width. The BF in support of H0 is depicted for the different tasks. The max BF H1 prior is the prior value in the range of *r* = 0–1.5 that gave the BF most favorable of H1.

Descriptive statistics for the odor TDI and the trigeminal and taste thresholds are plotted in Figure 1, with additional information provided in Table 1. We found no evidence in support of OC modifying odor detection thresholds. In fact, there was substantial evidence for the H0 that OC did not change odor thresholds, as indicated by a Bayesian two-sample *t* test (BF_01_ = 3.230, error % = 0.004). Similarly, odor discrimination was not affected by the use of OC with the result showing substantial evidence in support of H0 (BF_01_ = 3.628, error % = 0.004). For odor identification, the data were inconclusive, supporting neither H1, nor H0 (BF_01_ = 0.983, error % = 0.010). There was, however, substantial evidence that OC does not impact trigeminal thresholds (BF_01_ = 3.598, error % = 0.004). Likewise, OC does not impact taste thresholds (BF_01_ = 3.571, error % = 0.004). To provide a more detailed picture, the BFs across tasks are plotted with varying Cauchy prior scale from 0 to 1.5 in Figure 2. As evident from Figure 2, even when cherry-picking the most favorable prior for H1 (Max BF H1), there was only, at best, inconclusive evidence for H0 (Figure 2).

**Table 1.** Detailed results of the discrimination, identification, and threshold tests.

Group	Test	Stimuli	Score (CI 95)	Chemical threshold
NC	Discrimination	Sniffin' Sticks	11.74 (± 0.82)	-
OC	Discrimination	Sniffin' Sticks	12.00 (± 0.78)	-
NC	Identification	Sniffin' Sticks	32.15 (± 1.08)	-
OC	Identification	Sniffin' Sticks	30.73 (± 1.14)	-
NC	Threshold	Butanol	8.51 (± 0.76)	1.56 *10^-2^ %
OC	Threshold	Butanol	8.49 (± 0.84)	2.86 *10^-2^ %
NC	Threshold	Peanut	9.23 (± 0.65)	1.59 *10^-1^ %
OC	Threshold	Peanut	9.82 (± 0.87)	8.80 *10^-2^ %
NC	Threshold	Menthol	7.76 (± 0.99)	2.93 %
OC	Threshold	Menthol	7.49 (± 1.21)	4.39 %
NC	Threshold	Quinine	15.18 (± 1.54)	1.32 *10^-6^ M
OC	Threshold	Quinine	14.94 (± 1.53)	1.32 *10^-6^ M
NC	Threshold	Sucrose	8.96 (± 0.56)	1.39 *10^-2^ M
OC	Threshold	Sucrose	9.05 (± 0.59)	1.39 *10^-2^ M

*Note*. The chemical threshold column depicts the stimulus concentration that was the outcome of the threshold detection test. NC = normal cycling; OC = oral contraceptives.

**Figure 3.**
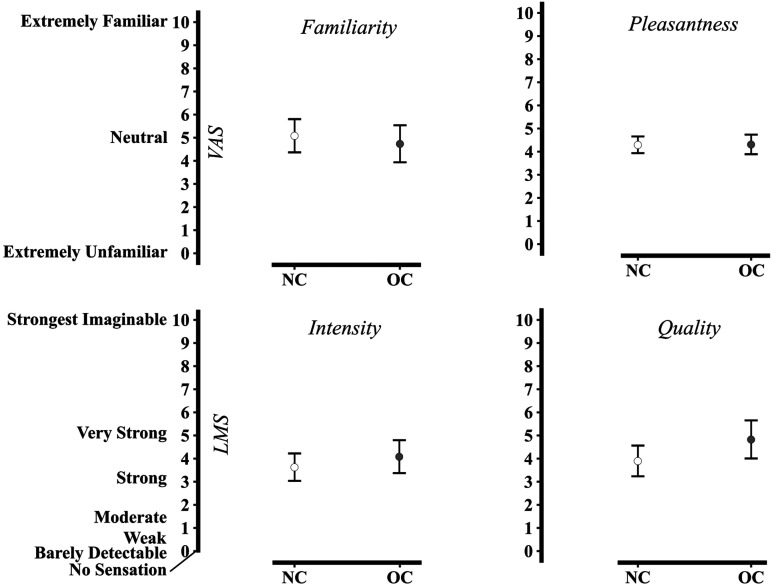
Average perceptual ratings of tastants. Participants rated the familiarity, pleasantness, intensity, and quality of sucrose and quinine. Error bars display 95% confidence interval. VAS = visual analogue scale; LMS = labelled magnitude scale; NC = normal cycling; OC = oral contraceptives.

Next, we wanted to determine whether there was a difference in perception of the tastants between nonusers of OC (NC) and OC users. Using a Bayesian ANOVA with taste perception test scores as the variable of interest, Group (OC vs. NC) and Dimension (familiarity, pleasantness, intensity, and quality) were treated as fixed factors and Subject as random factor (see Figure 3 and Table 2). The ANOVA favored the model including the dimensions (BF_10_ = 5.051, error % = 0.450), whereas the model including Group indicated that there are no group differences (BF_10_ = 0.259, error % = 0.963), and the models including Group and Dimension, as well as their interaction, produced inconclusive evidence (see Table 2). A post hoc test to investigate the Group effect of OCs resulted in a BF_01_ of 3.954 (error % = 1.932e^−5^), thus favoring the model without Group as an explanatory variable. The BF was corrected for multiple testing by fixing the prior probability that H0 holds across comparisons to 0.5 (Westfall et al., 1997).

**Table 2.** Bayesian Analysis of Variance (ANOVA) of the Perceptual Ratings of Tastants.

Model comparison for taste perception as function of OC					
Models	P(M)	P(M|data)	BF_M_	BF_10_	Error %
Null model (incl. Subject)	0.200	0.124	0.557	1.000	
Dimension	0.200	0.626	6.693	5.051	0.450
Group + Dimension	0.200	0.166	0.797	1.341	0.980
Group + Dimension + Group × Dimension	0.200	0.052	0.219	0.419	1.418
Group	0.200	0.032	0.133	0.259	0.963

*Note*. All models include Subject. P(M) indicates the prior probability assigned to the different models. The column P(M|data) displays the posterior model probabilities. The column BF_M_ indicates the degree to which the data have changed the prior models. The column BF_10_ shows the BF in favor of H1. OC = oral contraceptives; BF = Bayes factor.

## Discussion

Previous research investigating the effect OC on olfactory sensitivity has been inconclusive. Odor sensitivity has previously been shown to be both positively and negatively affected by pill use, positively and negatively affected by duration of pill intake, and potential differences seem to have been odor-dependent (Derntl et al., 2013; Kollndorfer et al., 2016; Lundström et al., 2006; Renfro & Hoffmann, 2013). Here, we revisited the question of whether OC use affects olfactory performance as well as assessed potential effects of OC use on trigeminal and taste perception. Our results indicate that OC use does not impact chemosensory perception. In fact, we considered a wide range of priors, and the null hypothesis (H0) was consistently favored over the alternative hypothesis (H1). Importantly, as trigeminal thresholds were not affected by OC, the mixed results from earlier research likely do not depend on differences in odor trigeminality of the odors used. Also, the only measure that indicated a potential effect of OC in our data was not a sensory function, but a measure that can be considered as more cognitive (cued odor identification). Notably, women have been shown to outperform men in odor identification—an effect not thought to be mediated by differences in olfaction but rather in general language abilities (Larsson, 1997, 2002; Larsson et al., 2005, 2014). A recent meta-analysis on the effect of OC on cognition further demonstrated that there is little evidence that OC impacts cognitive functions with consistent evidence only demonstrated for verbal memory (Warren et al., 2014). These findings indicate that any potential effect of OC on odor identification may be due to an impact on language functions and not olfactory function per se.

The current study has, however, weaknesses including a rather small sample size of participants in each group (OC users and nonusers), thus making it difficult to reach definite conclusions due to low statistical power. Post hoc power calculations revealed that we had 0.47 power to detect a medium effect size (0.5 d) at alpha level .05 (Faul et al., 2007). Another limitation is that the type, and dosage, of the hormonal contents of the used OC varied, and we could not control the duration of OC intake. Both the dosage of the ethinyl estradiol content, and the duration of OC use, have been suggested to alter how OC usage impacts olfactory sensitivity (Derntl et al., 2013; Kollndorfer et al., 2016). Future research should take this into account and also test a wider range of odorants, tastants, and trigeminal compounds. We tried to minimize the limitation in number of stimuli by including ecological relevant stimuli (food and non-food-associated odors) as well as use different chemical compositions (monomolecular odors and mixtures). Despite these short comings, we argue that our findings are strengthened by the following points. Earlier studies have only used frequentist statistics and were not able to test the strength of the H0 in instances when no differences were found (Dienes, 2014). Moreover, previous studies have found an effect of OC use on olfactory sensitivity when assessing very specific outcomes (e.g., the sensitivity for a specific odor, the influence of hormone dosage of the OC content, the duration of pill intake, which menstrual phase the women in the control group were in, etc.), with no general or broader effects consistently shown.

In summary, we conclude that an effect of OC use on chemosensory perception is unlikely and, if present, presumably of a small effect size with negligible ecological relevance. This should come as good news to OC users as based on these results there is no need to be concerned about altered chemosensory perception.
